# Donor Human Milk Implementation in a High-Mother’s-Own-Milk NICU: Impact on Feeding Progression and Clinical Outcomes in Extremely Low Birth Weight Infants

**DOI:** 10.3390/nu18162608

**Published:** 2026-08-10

**Authors:** Jacky Herzlich, Omer Less, Bar Frumer, Ronella Marom, Laurence Mangel, Dror Mandel

**Affiliations:** 1 Department of Neonatology, Dana Dwek Children’s Hospital, Tel Aviv Sourasky University Medical Center, Tel Aviv 6423906, Israel; 2 Gray Faculty of Medical and Health Sciences, Tel Aviv University, Tel Aviv 6997801, Israel

**Keywords:** donor human milk, mother’s own milk, extremely low birth weight, enteral feeding, parenteral nutrition, necrotizing enterocolitis

## Abstract

**Background**: Donor human milk (DHM) is recommended when the mother’s own milk (MOM) is unavailable for extremely low birth weight (ELBW) infants. However, the impact of DHM implementation on NICUs with already high human milk utilization remains incompletely characterized. **Objective**: We aimed to evaluate the association between DHM implementation and feeding practices, feeding progression, parenteral nutrition (PN) exposure, and major neonatal morbidities in ELBW infants. **Methods**: We conducted a retrospective before–after cohort study in a single tertiary NICU including 116 ELBW infants with feeding data through day 28 (58 pre-DHM; 58 post-DHM). Exploratory multivariable models evaluated factors associated with NEC and PN duration. **Results**: In the post-DHM epoch, enteral feeding was initiated earlier (2 vs. 2.5 days, *p* < 0.001) and full enteral feeding was achieved sooner (14 vs. 24.5 days, *p* < 0.001). PN duration was numerically shorter (17.5 vs. 27 days, *p* = 0.088). Feeding shifted from predominantly exclusive MOM to mixed MOM + DHM regimens, eliminating formula-only feeding. NEC, feeding intolerance, bloodstream infection, and mortality did not differ between epochs. Feeding intolerance was associated with NEC, whereas lower gestational age predicted longer PN duration; epoch was not independently associated with either outcome. **Conclusions:** In this high-MOM NICU, DHM implementation was associated mainly with earlier feeding progression and reduced formula reliance, without a measurable reduction in NEC.

## 1. Introduction

Extremely low birth weight (ELBW) neonates are at high risk for morbidity and mortality during their hospitalization in the neonatal intensive care unit (NICU) [1,2,3]. Among the most severe complications affecting this vulnerable population is necrotizing enterocolitis (NEC), a potentially life-threatening gastrointestinal disease characterized by intestinal inflammation and necrosis [4,5,6]. The clinical presentation of NEC ranges from mild forms that respond to conservative management to severe cases requiring surgical intervention, including bowel resection [7,8].

Human milk, whether administered via direct breastfeeding or as expressed maternal milk, has been consistently associated with a reduced risk of NEC compared to preterm formula [9,10,11,12]. In addition to its protective effect against NEC, mother’s own milk (MOM) is linked to lower incidences of late-onset sepsis and bronchopulmonary dysplasia due to its protective components [11,13]. However, MOM is not always available in adequate volumes. In such cases, the preferred alternative to formula feeding is pasteurized donor human milk (DHM), provided by certified human milk banks [9,14].

Evidence regarding the protective role of DHM remains evolving. A recent Cochrane review by Quigley et al. (2024) reported that preterm neonates receiving DHM had a 50% lower risk of NEC compared to those fed with formula [9]. In contrast, a meta-analysis by Silano et al. found no significant reduction in the incidence of surgical NEC with DHM use, highlighting ongoing uncertainties about its effectiveness in preventing the most severe forms of the disease [15].

Beyond NEC prevention, DHM may influence other aspects of neonatal health.

Unlike MOM, DHM typically consists of mature milk expressed weeks to months postpartum and undergoes pasteurization, which alters its composition. It generally contains lower levels of protein, fat, and bioactive components, including immunomodulatory proteins, human milk oligosaccharides, and beneficial microbiota, that are crucial for gut development and immune protection in preterm neonates [16,17,18]. These compositional differences may impact growth trajectories and gastrointestinal health.

In addition, given the high cost of DHM compared to formula, assessing its ability to reduce severe outcomes, particularly surgical NEC, is vital for informing clinical and economic decision-making [19].

The impact of DHM may differ substantially between NICUs depending on baseline feeding practices and formula exposure. While DHM has been widely evaluated as an alternative to formula feeding, considerably less is known about its implementation in NICUs where utilization of MOM is already high and formula exposure is relatively limited. In such settings, DHM may function primarily as a supplemental strategy that facilitates earlier enteral feeding while preserving a human milk-based feeding strategy. We therefore evaluated the association between DHM implementation and feeding practices, nutritional progression, growth, and neonatal outcomes among ELBW infants before and after implementation of DHM in our NICU. We hypothesized that DHM implementation would support earlier enteral feeding progression, reduce reliance on formula supplementation, and potentially decrease NEC risk while maintaining adequate growth and feeding tolerance.

## 2. Material and Methods

### 2.1. Participants

This study represents a predefined subgroup analysis of a previously described cohort of very low birth weight (VLBW) infants (birth weight < 1500 g or gestational age < 32 weeks) [20]. The present analysis included ELBW infants (birth weight < 1000 g) born between 1 January 2018 and 30 June 2023. DHM was introduced into routine clinical practice in August 2020, defining the pre-DHM and post-DHM epochs. To provide a standardized observation period focused on early nutritional adaptation, primary analyses were restricted to the first 28 days of life. Infants who died before 28 days of life were excluded from early feeding progression analyses but are reported separately in the study flow diagram and mortality analyses. This study was approved by the local Institutional Review Board (0172-22-TLV, approved on 4 August 2022), with a waiver for informed consent due to its retrospective design. It was conducted in accordance with Good Clinical Practice guidelines and the Declaration of Helsinki.

### 2.2. Data Collection

Maternal variables included antenatal steroid exposure (partial or complete course), pre-eclampsia, and gestational diabetes mellitus (GDM).

Neonatal baseline characteristics included gestational age (GA), birth weight (BW), sex, mode of delivery, surfactant administration, and small-for-gestational-age (SGA) status according to the Israeli birth weight reference charts of Dollberg et al. [21].

Nutritional variables collected during the first 28 days of life included age at initiation of enteral feeding, age at achievement of full enteral feeding, duration of parenteral nutrition (PN), age at regaining BW, feeding regimen, and percentage exposure to MOM, DHM, and formula.

Feeding regimens during the first 28 days were categorized as exclusive mother’s own milk, exclusive formula, mother’s own milk supplemented with formula, mother’s own milk supplemented with donor human milk, mother’s own milk supplemented with donor human milk and formula, or exclusive donor human milk. Full enteral feeding was defined as an enteral intake of ≥140 mL/kg/day.

Primary nutritional outcomes were age at initiation of enteral feeding, age at achievement of full enteral feeding, and duration of parenteral nutrition. Secondary outcomes included NEC, feeding intolerance, bloodstream infection, hypoglycemia, phototherapy exposure, IVH, mortality before day 28, and mortality before hospital discharge.

NEC was defined as Bell stage II or higher, and feeding intolerance was defined as gastric residuals ≥20% of the previous feeding volume. Symptomatic hypoglycemia was defined according to the unit’s age-specific glucose thresholds and corresponding clinical management protocol. Weight gain percentage from birth weight was calculated at 14 and 28 days of life. The 28-day observation window was selected a priori because it encompasses the period of greatest nutritional transition in ELBW infants, during which enteral feeding progression, establishment of feeding regimens, and dependence on parenteral nutrition are determined. This standardized observation period also minimizes bias related to differences in survival duration and hospitalization length while allowing consistent assessment of early feeding outcomes.

Our local protocol emphasizes early enteral feeding for preterm and low birth weight neonates, using MOM first, then DHM, and formula only if neither is available. Feeds are started within hours (6 h) of birth if the infant is clinically stable, advanced gradually to full intake (140–160 mL/kg/day) while tapering parenteral nutrition, and fortified with milk fortifier once feed volumes reached 60 mL/kg/day in neonates <32 weeks or <1500 g. Feed progression was withheld or individualized during periods of clinical instability according to attending neonatologist’s judgment.

DHM was obtained from the Magen David Adom Breast Milk Bank and processed according to the milk bank’s quality-control procedures using Holder pasteurization. DHM was available for eligible infants when MOM was insufficient and was fortified according to the same volume-based protocol used for MOM. Probiotics were not routinely administered during either study epoch. The general feeding and fortification approach remained broadly consistent throughout the study period, although individualized clinical decisions were made when infants were unstable.

### 2.3. Statistical Analysis

Continuous variables are summarized as mean ± SD or median [IQR] according to distribution; categorical variables are summarized as counts and percentages. Group comparisons between epochs used Mann–Whitney U tests for continuous variables, and χ^2^ or Fisher’s exact test for categorical variables, as appropriate. No imputation was performed. Analyses used complete-case data for each model, and the number of included cases is stated in each regression table. Exploratory multivariable logistic regression for NEC included study epoch, GA, BW per 100 g to improve interpretability, feeding intolerance, and day of feeding initiation. The study was not powered a priori for modest differences in NEC, surgical NEC, or mortality because it was retrospective. Exploratory multivariable linear regression for PN duration included study epoch, GA, SGA status, feeding intolerance, and day of feeding initiation. Model fit was summarized using Cox and Snell and Nagelkerke R^2^ for logistic regression and R^2^, adjusted R^2^, F-statistic, and model *p*-value for linear regression. Statistical significance was defined as *p* < 0.05. IBM SPSS Statistics for Windows, version 29, was used for statistical data analyses.

## 3. Results

**Figure 1:**
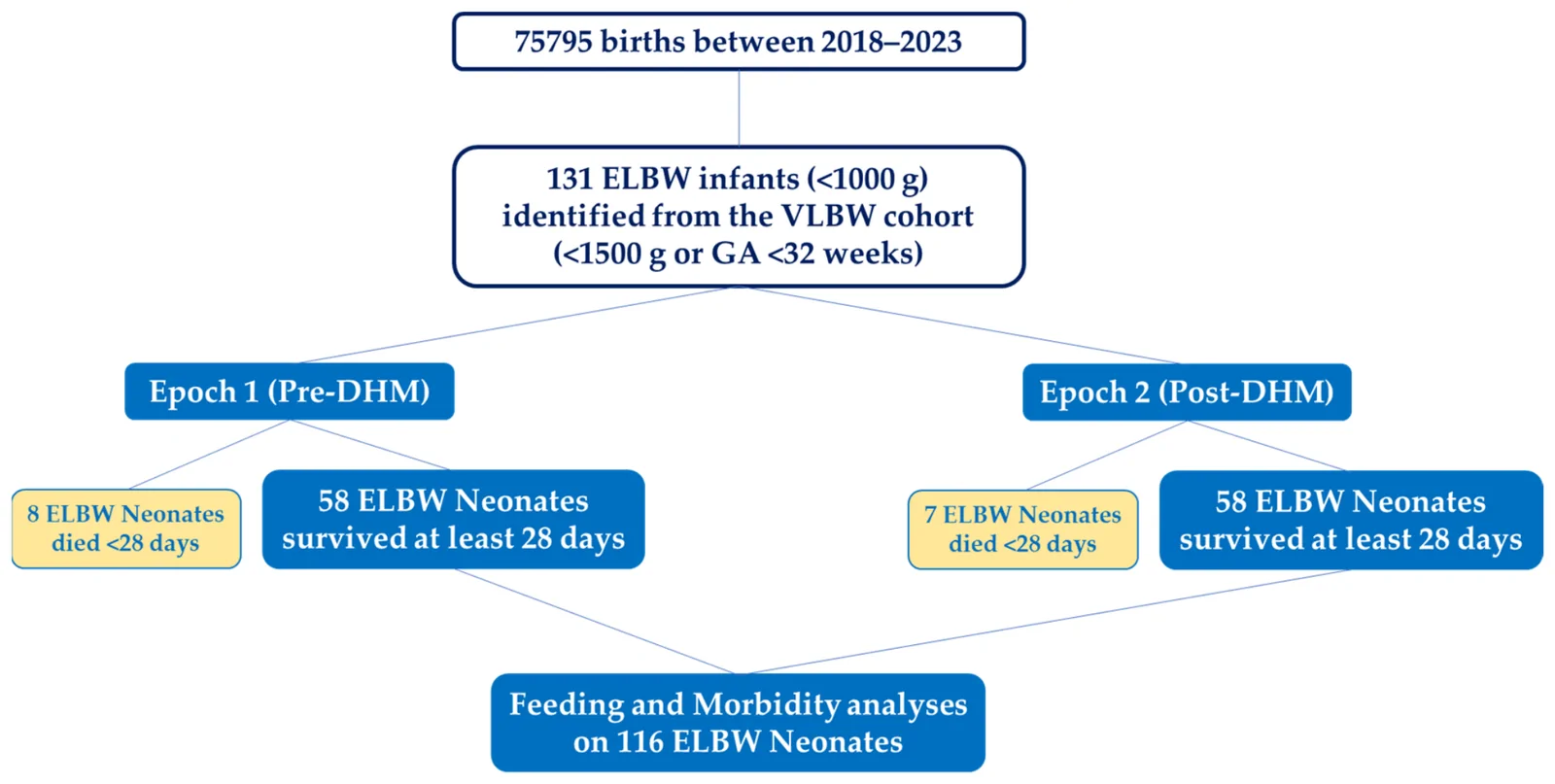
Study flow diagram of ELBM infants included before and after donor human milk implementation. Legend: Flow diagram showing identification of ELBW infants, allocation to pre-DHM and post-DHM epochs, deaths before day 28, and the final analytic cohort included in feeding and clinical outcome analyses.

A total of 131 ELBW infants were identified during the study period. Fifteen infants died before 28 days of life and were therefore excluded from the primary early feeding progression analyses. The final 28-day analytic cohort comprised 116 infants, including 58 infants in epoch 1 and 58 infants in epoch 2 (Figure 1).

### 3.1. Baseline Characteristics

Baseline characteristics are summarized in Table 1. GA, BW, sex, antenatal steroid exposure, pre-eclampsia, gestational diabetes, delivery mode, and hospital stay were similar between epochs, whereas infants were more frequently SGA in epoch 1 than in epoch 2 (55.2% vs. 29.3%, *p* = 0.005). Surfactant administration tended to be more frequent in epoch 1, although the difference did not reach statistical significance (75.9% vs. 60.3%, *p* = 0.073).

### 3.2. Feeding Practices and Nutritional Outcomes

Feeding practices and nutritional outcomes are presented in Table 2. Enteral feeding was initiated earlier in epoch 2 than epoch 1 (median 2 [1, 2] vs. 2 [2, 3] days, *p* < 0.001). Infants in epoch 2 achieved full enteral feeding substantially sooner than those in epoch 1 (median 14 [11,12,13,14,15,16,17,18,19,20,21,22] vs. 24.5 [14–35.5] days, *p* < 0.001).

Age at regaining BW did not differ between epochs (*p* = 0.316). Early growth was similar between epochs, with no significant differences in weight gain from birth weight at either 14 or 28 days. Median PN duration was shorter in epoch 2 (17.5 [12–27.5] vs. 27 [13.5–34] days), although the difference did not reach statistical significance (*p* = 0.088).

**Figure 2:**
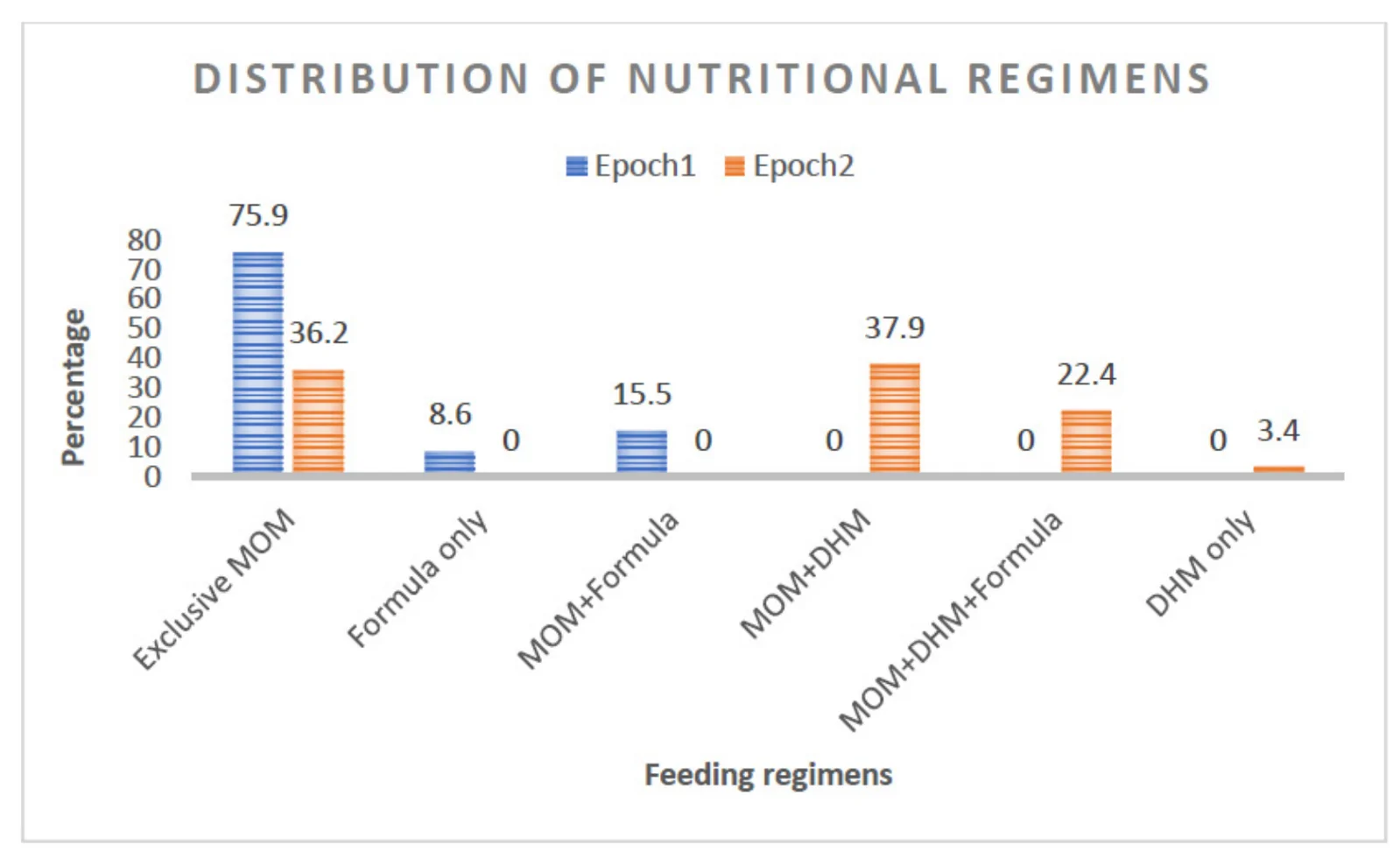
Distribution of feeding regimens during the first 28 days of life before and after donor human milk implementation. Legend: Following DHM implementation, feeding practices shifted from predominantly exclusive mother’s own milk feeding toward mixed mother’s own milk plus donor human milk regimens, while formula-only feeding was eliminated.

Feeding practices differed markedly between study epochs (Figure 2). Before DHM implementation, feeding regimens consisted predominantly of exclusive MOM feeding (75.9%), with limited use of formula alone (8.6%) or MOM supplemented with formula (15.5%). Following DHM implementation, exclusive MOM feeding declined to 36.2%, while mixed MOM + DHM regimens became the predominant strategy (37.9%). Additional infants received MOM + DHM + formula (22.4%) or exclusive DHM (3.4%), and formula-only feeding was eliminated. Despite these changes, MOM remained the predominant nutritional component of mixed feeding regimens.

### 3.3. Clinical Outcomes

Clinical outcomes are summarized in Table 3. NEC incidence did not differ significantly between epochs (25.9% vs. 20.7%, *p* = 0.510). Similarly, rates of feeding intolerance (31.0% vs. 29.3%, *p* = 0.840) and bloodstream infection (31.0% vs. 29.3%, *p* = 0.840) were comparable. Surgical NEC was uncommon in both epochs (5.2% vs. 8.6%). Rates of IVH were similar between epochs (29.3% vs. 41.4%, *p* = 0.174). Mortality before discharge did not differ significantly (6.9% vs. 8.6%, *p* = 0.728), nor did mortality for the whole ELBW cohort (*p* = 0.967). In contrast, symptomatic hypoglycemia was significantly more common in the post-DHM epoch (46.6% vs. 22.4%, *p* = 0.006). Phototherapy use was also significantly higher in epoch 2 (87.9% vs. 67.2%, *p* = 0.008).

### 3.4. Exploratory Multivariable Analyses

Exploratory multivariable analyses were performed to evaluate factors independently associated with NEC and PN duration (Table 4). In the logistic regression model for NEC, feeding intolerance was associated with NEC occurrence (aOR 2.60, 95% CI 1.04–6.49, *p* = 0.041), whereas study epoch, gestational age, birth weight, and day of feeding initiation were not. Model explanatory power was limited (Cox and Snell R^2^ = 0.066; Nagelkerke R^2^ = 0.100; omnibus χ^2^ = 7.91, *p* = 0.161) (Table 4).

In the multivariable linear regression model evaluating PN duration, lower gestational age independently predicted prolonged PN exposure (β = −3.67 days per week increase in GA, 95% CI −6.07 to −1.27, *p* = 0.003). Feeding intolerance demonstrated a borderline association with longer PN duration (β = 8.55 days, *p* = 0.062). Study epoch, SGA status, and day of feeding initiation were not independently associated with PN duration after adjustment. The overall model was statistically significant (R^2^ = 0.14, adjusted R^2^ = 0.10, F = 3.55, *p* = 0.005) (Table 4).

## 4. Discussion

The present observational before–after study evaluated implementation of DHM in a NICU already characterized by high utilization of MOM. Three principal findings emerged. First, the post-DHM epoch was associated with earlier initiation of enteral feeding and substantially faster achievement of full enteral nutrition. Second, implementation of DHM was accompanied by a marked shift in feeding strategies from predominantly exclusive MOM feeding toward mixed MOM + DHM regimens while eliminating formula-only feeding. Third, despite these changes in feeding practices and nutritional progression, major neonatal morbidities, including NEC, feeding intolerance, bloodstream infection, and mortality, remained similar between epochs.

Our findings suggest that, in a NICU already strongly committed to human milk feeding, the principal effect of DHM implementation was facilitation of early feeding progression rather than modification of major clinical morbidities. The post-DHM epoch was associated with earlier initiation and progression of enteral feeding while minimizing reliance on formula when maternal milk was temporarily unavailable. Earlier attainment of feeding milestones was not associated with impaired short-term growth, suggesting that routine fortification strategies were sufficient to maintain early growth despite increased DHM exposure. These findings support the role of DHM as a practical bridge when MOM is unavailable while reinforcing current recommendations that MOM remains the nutritional gold standard for preterm infants [9,14,22,23].

Contrary to our initial hypothesis, DHM implementation was not associated with a reduction in NEC incidence. This finding differs from randomized trials and meta-analyses demonstrating lower NEC rates among infants receiving DHM instead of formula [9,10,11,12]. The NEC incidence observed in our cohort was relatively high compared with several contemporary ELBW cohorts [24,25]. All NEC cases were individually reviewed and fulfilled the predefined diagnostic criteria. Differences in patient characteristics, referral patterns, and institutional practices may contribute to the variability in NEC incidence observed across centers. However, interpretation of those studies requires consideration of the baseline feeding environment. In many cohorts demonstrating NEC reduction, DHM largely replaced formula exposure. In contrast, infants in our unit already received predominantly human milk-based nutrition in the pre-DHM epoch. Consequently, DHM implementation may have had only a limited impact on overall human milk exposure. Recent discussions have emphasized that NEC risk may be influenced primarily by the protective effects of human milk and the relative absence of non-human milk feeding rather than by unique protective properties attributable specifically to donor milk [26,27]. This perspective may help explain the absence of a measurable NEC reduction in our cohort. Consistent with this interpretation, study epoch was not independently associated with NEC in our exploratory multivariable analysis. The study was not powered to detect modest differences in NEC, particularly surgical NEC.

An important observation was the marked decline in exclusive MOM feeding after DHM became available. Although most mixed feeding regimens in epoch 2 still contained a substantial proportion of MOM, fewer infants received nutrition exclusively from maternal milk. Similar patterns have been reported following DHM implementation and have prompted concerns that donor milk programs should complement rather than replace efforts to establish and maintain maternal milk supply [23,27,28,29]. Because maternal lactation variables were not measured, the reasons for this change cannot be determined from our data. Nevertheless, our findings reinforce the importance of maintaining active lactation-support programs whenever DHM is introduced. Importantly, in epoch 2, MOM remained the predominant component of mixed feeding regimens, with median MOM exposure of 82.5%, and no differences were observed in weight gain at either 14 or 28 days.

The higher prevalence of SGA in the pre-DHM epoch represents an important baseline imbalance. SGA status may influence nutritional tolerance, growth, and PN requirements; however, SGA was not independently associated with PN duration after adjustment, and early growth outcomes were similar between epochs. Nevertheless, residual confounding related to differences in fetal growth status cannot be excluded.

We also observed a higher incidence of hypoglycemia following DHM implementation. This unexpected finding should be interpreted cautiously because the retrospective design does not allow determination of causality, and unmeasured temporal changes in clinical practice may have contributed to the observed association. Nevertheless, donor milk differs from preterm MOM and exhibits substantial variability in nutrient composition, including lower protein and energy content [22,30,31]. In addition, earlier initiation of enteral feeding and greater reliance on DHM-supported feeding strategies may have contributed to differences in early metabolic adaptation in this vulnerable population. Although the underlying mechanisms remain uncertain, these findings highlight the importance of careful glucose monitoring and individualized nutritional management when DHM is used extensively, particularly among ELBW infants with limited metabolic reserves. Phototherapy use was more frequent following DHM implementation. This finding should be interpreted cautiously, as bilirubin concentrations, duration of treatment, and factors influencing bilirubin metabolism were not evaluated. The observed difference may reflect unmeasured differences in neonatal characteristics or clinical practice between study epochs rather than an effect of DHM itself. Accordingly, this finding should be considered exploratory and hypothesis-generating.

Taken together, these findings indicate that the clinical role of DHM in NICUs with already high MOM utilization differs from that reported in settings with greater baseline formula exposure, functioning primarily as a nutritional bridge that supports earlier establishment of human milk feeding rather than producing measurable reductions in NEC.

This study has several limitations. Its retrospective single-center before–after design precludes causal inference, may limit generalizability, and cannot exclude the influence of unmeasured secular changes. The sample size was modest for relatively infrequent outcomes such as NEC, surgical NEC, and mortality; therefore, the multivariable analyses should be considered exploratory. Finally, data on maternal lactation support and expressed milk volumes were not systematically available, and the nutritional composition of individual DHM batches was not recorded in the study dataset, limiting detailed assessment of milk exposure and its relationship with clinical outcomes.

## 5. Conclusions

In conclusion, in a NICU already characterized by high maternal milk utilization, DHM implementation was primarily associated with earlier feeding progression and reduced reliance on formula supplementation without measurable reductions in NEC or mortality. These findings suggest that, in similar settings, DHM may function primarily as a nutritional bridge, facilitating earlier establishment of enteral feeding while preserving a human milk-based feeding strategy. Future multicenter prospective studies with larger cohorts and longer follow-up are warranted to confirm these findings and evaluate longer-term nutritional and clinical outcomes.

## Figures and Tables

**Table 1:** Maternal and neonatal characteristics of ELBW infants before and after donor human milk implementation.

Characteristics	Epoch 1 (*n* = 58)	Epoch 2 (*n* = 58)	*p*-Value
Antenatal steroid	40 (69)	46 (79.3)	0.203
Pre-eclampsia	11 (19)	10 (17.2)	0.844
Gestational diabetes mellitus	4 (6.9)	6 (10.3)	0.508
Mode of delivery			0.281
Spontaneous vaginal delivery	9 (15.5)	17 (29.3)	
Emergency cesarean	48 (82.8)	40 (69)	
Elective cesarean	1 (1.7)	1 (1.7)	
Gestational age (weeks)	27.1 ± 2.1 (24–32)	26.6 ± 2 (23–32)	0.231
Birth weight (g)	827.5 [698.8, 932.5] (500–995)	827.5 [732.5, 980] (550–996)	0.478
Female sex	30 (51.7)	28 (48.3)	0.710
Small for gestational age	32 (55.2)	17 (29.3)	**0.005**
Surfactant use	44 (75.9)	35 (60.3)	0.073
Length of hospital stay (d)	82 [55.8, 108.3] (40–224)	83 [67.8, 105] (30–197)	0.952

Data are presented as mean ± SD (range), median [Q1, Q3] (range), or *n* (%). Continuous variables were compared using Mann–Whitney U tests and categorical variables using χ^2^ or Fisher’s exact tests. Significant *p*-values are shown in bold.

**Table 2:** Feeding practices, nutritional progression, and growth outcomes before and after donor human milk implementation in ELBW infants.

Characteristics	Epoch 1 (*n* = 58)	Epoch 2 (*n* = 58)	*p*-Value
Start of enteral feeding (d)	2.5 ± 1 2 [2, 3]	2 ± 2.6 2 [1, 2]	**<0.001**
Reaching birth weight (d)	8 [5.8, 12]	9.5 [6, 14]	0.316
Duration of parenteral nutrition (d)	27 [13.8, 35.8]	17.5 [12, 27.5]	0.088
Reaching full enteral feeding (d)	24.5 [14, 35.5]	14 [11, 22]	**<0.001**
WG% at 14 days	6.3 [−1.3, 17]	8.4 [1, 14]	0.903
WG% at 28 days	33.7 [17.3, 52.6]	37.2 [23.1, 46.7]	0.862
Feeding regimen			**<0.001**
MOM	44 (75.9)	21 (36.2)	
Formula	5 (8.6)	0	
MOM + formula	9 (15.5)	0	
MOM + DHM	0	22 (37.9)	
MOM + DHM + formula	0	13 (22.4)	
DHM	0	2 (3.4)	
Exclusive MOM rate (%)	44 (75.9)	21 (36.2)	**<0.001**
MOM exposure in mixed feeding regimen (% of feeds)	75 [65, 87.5]	82.5 [58, 90]	0.775
Formula exposure in mixed feeding regimen (% of feeds)	25 [12.5, 35]	14 [7.5, 28.5]	0.144
DHM use (% of feeds)	0	10 [7, 32]	NA

Data are presented as mean ± SD, median [Q1, Q3] or *n* (%). *p*-Values were calculated using Mann–Whitney U tests for continuous variables and χ^2^ or Fisher’s exact tests for categorical variables. MOM, mother’s own milk; DHM, donor human milk; WG%, weight gain percentage from birth weight; NA, not assessed. Significant *p*-values are shown in bold.

**Table 3:** Clinical outcomes and mortality before and after donor human milk implementation in ELBW infants.

Outcome	Epoch 1	Epoch 2	*p*-Value
Clinical Outcomes (28-day feeding cohort; *n* = 58 per epoch)			
Total necrotizing enterocolitis (NEC)	15 (25.9)	12 (20.7)	0.510
Surgical NEC	3 (5.2)	5 (8.6)	0.717
Feeding intolerance	18 (31)	17 (29.3)	0.840
Bloodstream infection	18 (31)	17 (29.3)	0.840
Intraventricular hemorrhage (IVH)	17 (29.3)	24 (41.4)	0.174
Symptomatic hypoglycemia	13 (22.4)	27 (46.6)	**0.006**
Phototherapy	39 (67.2)	51 (87.9)	**0.008**
Mortality before discharge	4 (6.9)	5 (8.6)	0.728
Mortality (full ELBW cohort; epoch 1 *n* = 66, epoch 2 n = 65)			
Mortality before day 28	8 (12.1)	7 (10.8)	0.808
Total mortality rate	12 (18.2)	12 (18.5)	0.967

Data are presented as *n* (%). Clinical outcomes were analyzed in the 28-day feeding cohort (*n* = 58 per epoch). Mortality outcomes were analyzed in the entire ELBW cohort (N = 131, epoch 1 *n* = 66, epoch 2 *n* = 65). Significant *p*-values are shown in bold.

**Table 4:** Exploratory multivariable analyses of necrotizing enterocolitis (Panel A) and parenteral nutrition duration (Panel B) in ELBW infants.

A. Exploratory Multivariable Logistic Regression Analysis for NEC (*n* = 116)			
Variable	Adjusted OR	95% CI	*p*-Value
Epoch 2 vs. epoch 1	0.76	0.30–1.93	0.569
Gestational age (weeks)	0.94	0.70–1.25	0.661
Birth weight (per 100 g)	0.88	0.57–1.35	0.555
Feeding intolerance	2.6	1.04–6.49	**0.041**
Day of feeding initiation	1.09	0.86–1.39	0.477
Model statistics: Cox and Snell R^2^ = 0.066; Nagelkerke R^2^ = 0.100; omnibus χ^2^ = 7.91 (df = 5), *p* = 0.161.			
**B. Multivariable Linear Regression Analysis for Parenteral Nutrition Duration (*n* = 116)**			
**Variable**	**β (days)**	**95% CI**	***p*** **-Value**
Gestational age (weeks)	−3.67	−6.07 to −1.27	**0.003**
SGA	−1.76	−11.23 to 8.73	0.805
Feeding intolerance	8.55	−0.42 to 17.52	0.062
Day of feeding initiation	−0.50	−2.61 to 1.61	0.640
Epoch 2 vs. epoch 1	−5.39	−13.88 to 3.10	0.211
Model statistics: N = 116, R^2^ = 0.14, adjusted R^2^ = 0.10, F = 3.55, *p* = 0.005.			

Data are presented as adjusted odds ratios (aORs) or β coefficients with 95% confidence intervals (CIs). Birth weight was entered into regression models per 100 g increment. SGA, small for gestational age. Significant *p*-values are shown in bold.

## Data Availability

The data presented in this study are available on request from the corresponding author due to their containing information that could compromise the privacy of research participants.
